# Partial Discharge Localization Using Time Reversal: Application to Power Transformers

**DOI:** 10.3390/s20051419

**Published:** 2020-03-05

**Authors:** Hamidreza Karami, Mohammad Azadifar, Amirhossein Mostajabi, Marcos Rubinstein, Hossein Karami, Gevork B. Gharehpetian, Farhad Rachidi

**Affiliations:** 1 Electromagnetic Compatibility Laboratory, Swiss Federal Institute of Technology (EPFL), 1015 Lausanne, Switzerland; amirhossein.mostajabi@epfl.ch; 2 Department of Electrical Engineering, Bu-Ali Sina University, 65178 Hamedan, Iran; 3 University of Applied Sciences of Western Switzerland (HES-SO), 1400 Yverdon-les-Bains, Switzerland; mohammad.azadifar@heig-vd.ch (M.A.); rubinstein.m@gmail.com (M.R.); 4 High-Voltage Research Group, Niroo Research Institute, 14665-517 Tehran, Iran; hkarami@nri.ac.ir; 5 Electrical Engineering Department, Amirkabir University of Technology (Tehran Polytechnic), 15914 Tehran, Iran; grptian@aut.ac.ir

**Keywords:** time-reversal, partial discharge, source localization, insulation dielectric, power transformers

## Abstract

In this work, we present a novel technique to locate partial discharge (PD) sources based on the concept of time reversal. The localization of the PD sources is of interest for numerous applications, including the monitoring of power transformers, Gas Insulated Substations, electric motors, super capacitors, or any other device or system that can suffer from PDs. To the best of the authors’ knowledge, this is the first time that the concept of time reversal is applied to localize PD sources. Partial discharges emit both electromagnetic and acoustic waves. The proposed method can be used to localize PD sources using either electromagnetic or acoustic waves. As a proof of concept, we present only the results for the electromagnetic case. The proposed method consists of three general steps: (1) recording of the waves from the PD source(s) via proper sensor(s), (2) the time-reversal and back-propagation of the recorded signal(s) into the medium using numerical simulations, and (3) the localization of focal spots. We demonstrate that, unlike the conventional techniques based on the time difference of arrival, the proposed time reversal method can accurately localize PD sources using only one sensor. As a result, the proposed method is much more cost effective compared to existing techniques. The performance of the proposed method is tested considering practical scenarios in which none of the former developed methods can provide reasonable results. Moreover, the proposed method has the unique advantage of being able to locate multiple simultaneous PD sources and doing so with a single sensor. The efficiency of the method against the variation in the polarization of the PDs, their length, and against environmental noise is also investigated. Finally, the validity of the proposed procedure is tested against experimental observations.

## 1. Introduction

Nowadays, power transformers play a key role in electrical power system grids. Any failure in power transformer functionality would decrease the reliability of electrical power networks. Partial Discharges (PDs) are partial electrical breakdowns taking place in transformer insulation. PDs may result, in the long term, in the breakdown of the insulation system and severe damage to the transformer. Therefore, the detection and localization of PD sources are important diagnostic tools to monitor the insulation condition in power transformers.

PD measurement techniques [1] can be divided into four categories, (1) electrical, (2) chemical, (3) acoustic, and (4) electromagnetic. 

In the electrical detection category [1,2] which is based on IEC 60270, the current or voltage waveforms are measured at the high voltage and low voltage terminals of the power transformer. The high frequency content of the signals is used to estimate the existence of PDs. The methods belonging to the electrical category allow the determination of the apparent charge associated with the PD. They are also sensitive to weak PD activity and they are mainly useful only for the detection of PDs inside the transformer, even though some of the methods in this category can predict the turn number in which a PD occurred and only perform 1-D localization [3]. 

In chemical detection methods [1,4], the composition of dissolved gases in power transformer oil is analyzed to detect PDs. These methods cannot be applied to localize PDs in power transformers. 

Acoustic detection methods [1,5,6,7,8] are based on the detection of the sound waves emitted from the PD sources. Using these methods, the 3-D localization of PD sources is possible. However, the acoustic method has less sensitivity to weak PDs and those that occur inside the winding [5,9]. Acoustic sensors can be mounted on the outside walls of the power transformer, thereby making acoustic detection a non-invasive technique. However, the acoustic signal might be contaminated by the external acoustic environmental noise. On the other hand, the acoustic-based methods are not affected by electromagnetic interference in the measurement environment.

Electromagnetic detection methods [1,5,7,9,10,11] are applied to detect the electromagnetic waves radiated from the PD sources. Using these methods, the 3-D localization of PD sources is possible. The PD detection methods using Ultra High Frequency (UHF) radiation (UHF-PD) are sensitive to weak PDs and those that are inside the winding. In addition, UHF-PD measurements are usually electromagnetically shielded by the grounded transformer tank against external disturbances like corona and environmental noise. 

Classical acoustic and electromagnetic 3D-localization methods are based on the time difference of arrival (TDoA) of signals. These methods are highly sensitive to noise since TDoA relies on a precise determination of the onset time of the arriving signals [12]. They also need at least four time-synchronized sensors to operate. The TDoA-based techniques in the acoustic methods can provide reasonable accuracy by performing proper signal processing and having suitable propagation paths from the PD sources to the multiple sensors. However, TDoA-based electromagnetic methods suffer from inaccuracies because of inhomogeneities and scattering inside transformers.

**Figure 1:**
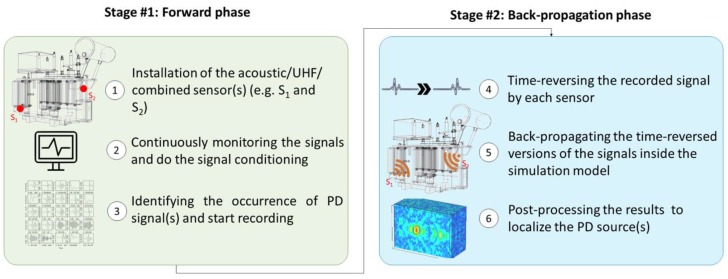
Schematic view of the proposed partial discharge (PD) localization approach based on the TR method.

This paper proposes a new method based on time reversal to locate PD sources in power transformers using either acoustic or electromagnetic sensors. The proposed method is able to locate PD sources in a 3D volume with less than four sensors. The proposed procedure is shown schematically in Figure 1.

One or more acoustic, UHF, or combined sensors are installed inside the power transformer tank or, alternatively, one or more acoustic sensors are installed on the exterior wall of the tank. The signals from a single or from multiple PDs are continuously monitored and processed as needed (e.g., filtered, denoised, amplified, etc.), digitized, and relayed to an industrial computer or some other processing unit. The occurrence of the PD signals is identified by a suitable criterion for, for example, the acquired signals crossing of a threshold value. 

The signals from the previous stage are time reversed and back propagated into the acoustic/electromagnetic model of the transformer tank. It should be noted that this step is performed in a simulation environment. In order to obtain the location of the PD source(s) in the transformer tank, the maximum electric/acoustic field criterion is employed.

The novelties of this work and its advantages with respect to existing technologies can be listed as follows:

A—The Time Reversal (TR) method [13] is used for the first time to localize PD sources.

B—Current state-of-the-art PD localization methods such as TDoA require at least four sensors to perform. In contrast, the proposed method is able to reduce the number of required sensors for the localization of PD sources to less than four sensors and it can perform well even with a single sensor.

C—The proposed method shows excellent localization skill in the case of multiple PD sources inside the test object. In fact, the numerical simulations (see Section 3) show that multiple PD sources can be precisely localized with only one UHF probe. Current technologies are incapable of locating multiple simultaneous PD sources.

D—In the proposed method, the location accuracy is not affected by the signal onset times and it is better than λ/10 in the worst case, where λ is the wavelength at the upper cutoff frequency of the signal. The numerical results confirm that the maximum error to localize the PD sources using UHF probes is less than 8 mm, which is lower than λ/10.

The proposed technique is tested using a computer simulation of a power transformer. The transformer is modelled representing the tank as a cavity. Three cylindrical tubes are used to represent the windings. Multiple PD sources are generated using dipole antennas in this model and the UHF signal from them is captured using a single dipole antenna in the tank. The effect of environmental noise is investigated by mixing the acquired signal with Gaussian white noise. The received signal is time reversed and back propagated into the medium and the maximum electric field criterion is used to locate the 3D position of each individual PD source. The rest of the paper is organized as follows. In Section 2, we describe the proposed method including the theoretical background and mathematical derivation. In Section 3, we present simulation results, including various scenarios to validate the proposed method and verify its accuracy. Section 4 presents a summary and conclusions.

## 2. Methodology

Since PDs produce acoustic and electromagnetic radiation, the problem of localizing PDs within the tank of a transformer can be considered equivalent to that of the localization of acoustic or electromagnetic sources. In this section, we present the concept and application of time reversal for electromagnetic waves. Similar principles can be derived for acoustic waves.

### 2.1. Principle of Electromagnetic Time Reversal

We use here the time reversal invariance property of Maxwell’s equations in the soft sense [13]. By setting *t* → -*t* in Maxwell’s equations, one can write:

(1) $$\begin{array}{l} \nabla \cdot \left(\varepsilon (\vec{r})\vec{E}(\vec{r},\;-t)\right)=\rho (\vec{r},\;-t)\\ \nabla \cdot \left(\mu (\vec{r})\;(-\vec{H}(\vec{r},\;-t))\right)=0\\ \nabla \times \vec{E}(\vec{r},\;-t)=-\mu (\vec{r})\frac{\partial (-\vec{H}(\vec{r},\;-t))}{\partial t}\\ \nabla \times (-\vec{H}(\vec{r},\;-t))=\varepsilon (\vec{r})\frac{\partial \vec{E}(\vec{r},\;-t)}{\partial t}+(-\vec{J}(\vec{r},\;-t)) \end{array}$$

It can be seen that if we reverse time, Maxwell’s equations still hold under the condition of changing the sign of the magnetic field and the current density (i.e., $\vec{H}(\vec{r},t)\to -\vec{H}(\vec{r},-t)$ and $\vec{J}(\vec{r},t)\to -\vec{J}(\vec{r},-t)$). These sign changes can be explained by the fact that, when the direction of time is reversed, the velocity of the charges changes sign and, as a consequence, so does the sign of the electrical current and of the associated magnetic field [13]. 

Now, let us assume a lossless medium in which there is at least one source emitting waves. If we record the emitted waves using a sufficient number of sensors, the time reversal nature of the governing wave equation guarantees that reinjected time-reversed versions of the recorded waves will refocus back to the primary source location or locations. In practical situations, the quality of the far-field focusing that can be obtained depends on the number of recording sensors and on the losses in the propagation medium. Moreover, it has been experimentally proven that the efficiency of the time reversal process improves in highly scattering media, in which the focusing property of time reversal can be better exploited via multipath propagation between sources and sensors [14].

In the case of PD sources in a transformer, the transformer tank can be considered as an enclosed cavity. The focusing property of time reversal in cavities has been mathematically and experimentally established by [15,16]. Neglecting losses on the walls of the transformer tank and other materials within the tank, one can expect that the strong focusing property of time reversal in cavities can be exploited to localize PD sources.

Three steps must be taken in order to locate sources via the time reversal process:

(*i*) The electromagnetic or acoustic waves from the source or sources are measured or calculated (forward-time) at one or multiple locations.
(*ii*) The acquired waveforms are time-reversed and back-injected into the solution medium, using numerical simulations (backward-time).
(*iii*) A criterion to detect and locate the source(s) is applied within the backward-time phase, such as the maximum amplitude of the total wave, its maximum energy, entropy, etc., to obtain the focal spot.

In this paper, we will use two criteria for the third step, namely the maximum field amplitude [17] and the entropy [18,19]. The entropy method makes use of the fact that the spatial distribution of the field is characterized by a minimum entropy at the time at which the time-reversed back-injected waves reach the source [18,19]. The focusing time is first determined by evaluating the entropy of the field as a function of time. The last local minimum of the entropy corresponds to the focusing time. The location of the source is determined by examining the spatial distribution of the electric field at the focusing time: the maximum value corresponds to the source location.

### 2.2. Mathematical Derivation

Herein, we assume the transformer tank as a rectangular box. The dyadic Green’s function in Cartesian coordinates of the magnetic vector potential for a rectangular cavity with dimensions of *a* × *b* × *c* can be written as:

(2) $${\overset{↔}{G}}_{A}=G_{A}^{xx}\hat{x}\hat{x}+G_{A}^{yy}\hat{y}\hat{y}+G_{A}^{zz}\hat{z}\hat{z}$$

Let us assume that the PD source is an infinitesimal dipole source in the direction of the *y*-axis at point (*x*, *y*, *z*) and derive $G_{A}^{yy}$ as presented in Equations (3a) and (3b) using the concept of image theory. Other components of ${\overset{↔}{G}}_{A}$ can be written accordingly:

(3a) $$G_{A}^{yy}=\frac{\mu_{0}}{4\pi }\sum_{m=-\infty }^{\infty }\sum_{n=-\infty }^{\infty }\sum_{p=-\infty }^{\infty }\sum_{i=1}^{8}S_{i}\frac{e^{-jkR_{i,mnp}}}{R_{i,mnp}}$$

where *µ*_0_ is the free-space permeability and

(3b) $$R_{i,mnp}=\sqrt{{\left(X_{i}-2ma\right)}^{2}+{\left(Y_{i}-2nb\right)}^{2}+{\left(Z_{i}-2pc\right)}^{2}}$$

in which, $X_{i}=\left|x-x_{i}^{\prime }\right|$, $Y_{i}=\left|y-y_{i}^{\prime }\right|$, $Z_{i}=\left|z-z_{i}^{\prime }\right|$, where $\left(x_{i}^{\prime },y_{i}^{\prime },z_{i}^{\prime }\right)$ is the location of the image source and *Si* depends on the orientation of the image source and the sign of the current, *µ*_0_ is the permeability of free space, and *k* is the wavenumber. 

The electric field at the observation point can be found by the electric field integral equation as [20]:

(4) $$\vec{E}(r,\omega )=\frac{-j\omega }{4\pi }\int_{l}\left[\overset{↔}{I}+\frac{\nabla \nabla }{k^{2}}\right]{\overset{↔}{G}}_{A}(r,r^{\prime },\omega ).\vec{J}(r^{\prime },\omega )dr^{\prime }$$

where, *ω*, $\overset{↔}{I}$, r, and $r^{\prime }$ are the angular frequency, the unit dyadic, and the observation and source vectors, respectively.

We have derived the electric field in the forward time step at the sensor location. In the next step, the time reversed field can be obtained by:

(5) $$\begin{array}{l} {\vec{E}}^{TRC}(r,\omega )={\left(\frac{\omega }{4\pi }\right)}^{2}\int_{l}\int_{l^{\prime }}\left(\overset{↔}{I}+\frac{\nabla \nabla }{k^{2}}\right)\overset{↔}{G}(r,r^{\prime },\omega ).\\ (\overset{↔}{I}+\frac{\nabla \nabla }{k^{2}}){\overset{↔}{G}}^{*}(r^{\prime \prime },r^{\prime },\omega ).{\vec{J}}^{*}(r^{\prime \prime },\omega )dr^{\prime }dr^{\prime \prime } \end{array}$$

In (5), the raised asterisk denotes the complex conjugate.

### 2.3. Numerical Model

The transient solver of the CST Microwave Studio software [21] is used to simulate the electromagnetic wave propagation inside the transformer tank. This solver uses the Finite Integration Technique (FIT) to solve the Maxwell’s equations in their integral form. It should be noted that both the forward and backward steps of the time reversal process are implemented in the CST software. In the forward propagation step, we use a Gaussian pulse to excite the structure, while in the backward propagation step, the time-reversed signal obtained in the former step is imported into CST. The backward propagation step is similar to the forward step except for the excitation pulse. 

The CST model has been validated using an experimental setup. The result is provided in Section 3.5.

## 3. Proof of Concept: Simulations and Results

**Figure 2:**
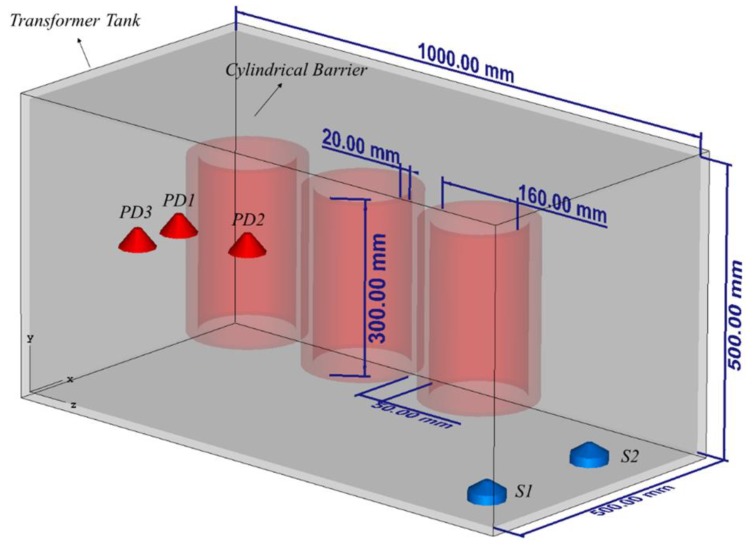
The geometry of the problem used in case studies CS #1-5 (see Table 1).

To prove the feasibility and the performance of the method, we have designed and performed several case studies listed in Table 1. Figure 2 shows the transformer tank, including three metallic cylinders as the windings. The materials of the transformer tank and the windings are steel (σ = 7.69e6 S/m) and copper (σ = 5.8e7 S/m), respectively. The thickness of the transformer tank is assumed to be 10 mm. The distance between the cylinders is 50 mm and between the cylinders and the wall of the tank is 150 mm. Other related parameters are provided in Figure 2.

Four PD sources are modeled as dipole antennas excited with a Gaussian pulse with frequency bandwidth of 300–3000 MHz [16], as shown in Figure 2. These sources are located in such a way that they represent specific conditions when the PD occurs outside, inside, and between the windings. Two UHF probes are placed inside the tank. As an example, they have been assumed to be located on the bottom of the transformer (see Figure 2). In all case studies, the length of the UHF probes is 20 mm. The details of the location of the considered sensors and PD sources can be seen in Table 2.

CS #1 aims to prove the feasibility of the TR method in localizing a PD source inside an empty transformer tank. The study then continues with CS #2 to prove that the TR method requires only one sensor to accurately localize a PD source inside the transformer tank. We further included three active parts and applied the proposed approach (CS #3). Finally, CS #4-5 prove that the excellent performance of the TR method remains intact when multiple PD sources occur simultaneously inside the test object. What follows explains the simulation results of each of the aforementioned case studies.

### 3.1. Capability of the TR Method to Localize a PD Source (CS #1)

**Figure 3:**
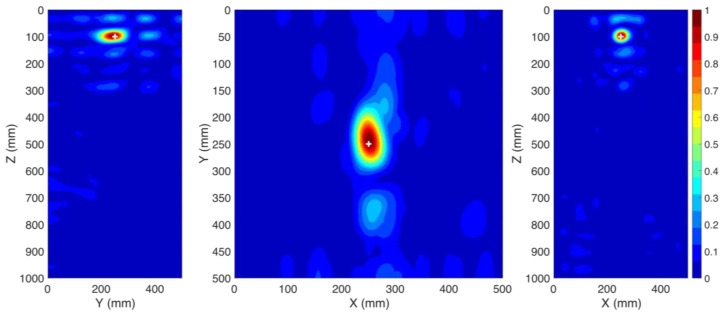
Normalized distribution of the maximum electric field power over the time inside the transformer tank (CS #1). The ground truth location of the PD source (PD1) is shown by the “+” marker. Two sensors (S1 and S2) are used.

In this case study, the windings are excluded from the presented transformer in Figure 2. The coordinates of the location of the PD source are those of PD1 according to Table 2. In this case, both sensors (S1 and S2) are used with their locations provided in Table 2. The method depicted in Figure 1 is applied. Figure 3 presents the distribution of the maximum electric field power in three cut planes within the transformer tank over the total simulation time. The three cut-planes correspond to the position of the overall maximum electric field in time and space. The point corresponding to the maximum electric field power in Figure 3 is estimated as the location of the PD source by the proposed method. The ground truth location of the PD source (PD1) is shown by the “+” marker. The 3D location error is estimated to be 6 mm, which is smaller than λ/10, where λ is equal to 100 mm at the upper cutoff frequency of the signal [22]. This result shows that the TR method can be effectively used to localize a PD source inside the transformer tank. To the best of the authors’ knowledge, this is the first proof of concept of the applicability of the TR method to locate PD sources with less than three UHF sensors.

### 3.2. Single Sensor PD Localization (CS #2)

**Figure 4:**
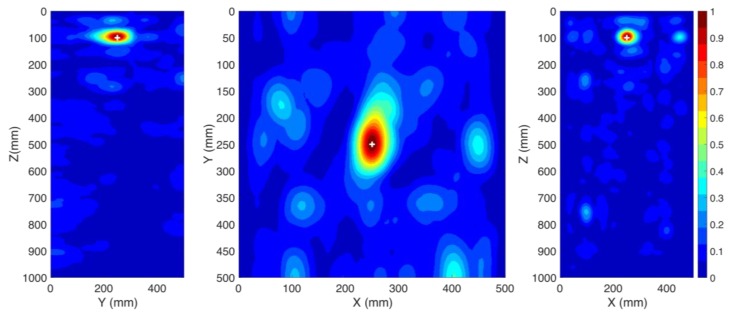
Normalized distribution of the maximum electric field power over the time inside the transformer tank (CS #2). The ground truth location of the PD source (PD1) is shown by the “+” marker. Only one sensor (S1) is used.

In this section, we investigate the performance of the proposed method when only one sensor is used. The location of the PD source remained the same as in CS #1. In this case, sensor S1 is kept operational and sensor S2 is removed. The windings are excluded from the transformer presented in Figure 2 and the method described in Section 2 is applied. The obtained results are depicted in Figure 4. The 3D location error is estimated to be 5 mm, which is smaller than λ/10, where λ is equal to 100 mm (corresponding to the upper cutoff frequency of the signal). As it can be observed, the proposed method can successfully locate the PD source using only one sensor and its accuracy did not degrade by reducing the number of sensors from two to one.

### 3.3. Single Sensor PD Localization in the Presence of the Active Parts (CS #3)

**Figure 5:**
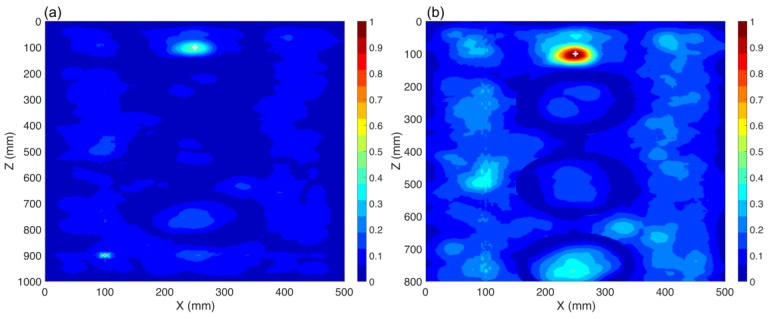
Normalized distribution of the maximum electric field power over the whole simulation time inside the transformer tank in the x-z cut plane (CS #3). The active parts of the transformer are included. The ground-truth location of the PD source (PD1) is shown by the “+” marker. Only one sensor (S1) was used. (**a**) full x-z cut plane, (**b**) x-z cut plane removing the area around the sensor.

So far, the transformer tank has been modelled as an empty cavity in the simulations. However, the presence of the active parts changes the wave propagation inside the tank, which may affect the localization results [17]. Here, we include the three simple copper cylinders shown in Figure 2, representing windings of the transformer (active parts). The location of the PD source (PD1) and the sensor (S1) remained the same as in CS #2. It should be noted that the location of PD1 is behind the active part of the transformer and there is no line of sight path to the sensor, a scenario which is quite challenging for the TDoA approach. The method in Figure 1 is applied again in this case. The obtained results in the x-z cut plane are shown in Figure 5. Figure 5a shows the distribution of the maximum electric field power over the whole time. According to Figure 5a, in contrast to CS #1 and CS #2, the overall maximum over the whole time and space coincides with the location of the sensor. However, the primary knowledge of the location of the sensor is available and that location can therefore be disregarded. The second overall maximum corresponds to the location of the PD source (PD1). Figure 5b shows the x-z cut plane by removing the area of the known sensor. As shown in that figure, the locations of the winding cylinders are marked with close to zero fields. The 3D location error is estimated to be 4 mm, which, as in all the previous cases, is smaller than λ/10, where λ is equal to 100 mm (corresponding to the upper cutoff frequency of the signal). The proposed method can successfully locate the PD source even by including the active parts of the transformer. In other words, the presence of large scatterers such as windings does not degrade the performance of the method.

### 3.4. Localization of Multiple PD Sources using the TR Method (CS #4-5)

**Figure 6:**
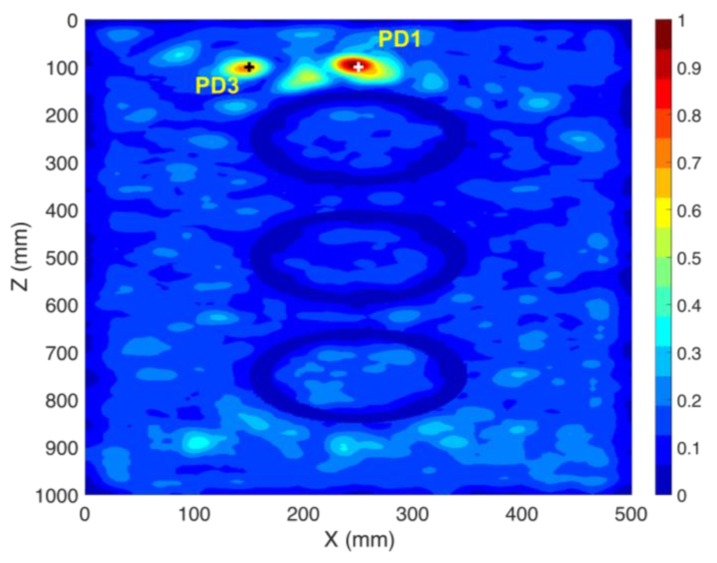
Normalized distribution of the maximum electric field power over the whole simulation time inside the transformer tank in the x-z cut plane (CS #4). The active parts of the transformer are included. The ground truth locations of the PD sources (PD1 and PD3) are shown by “+” markers. Only one sensor (S1) is used.

**Figure 7:**
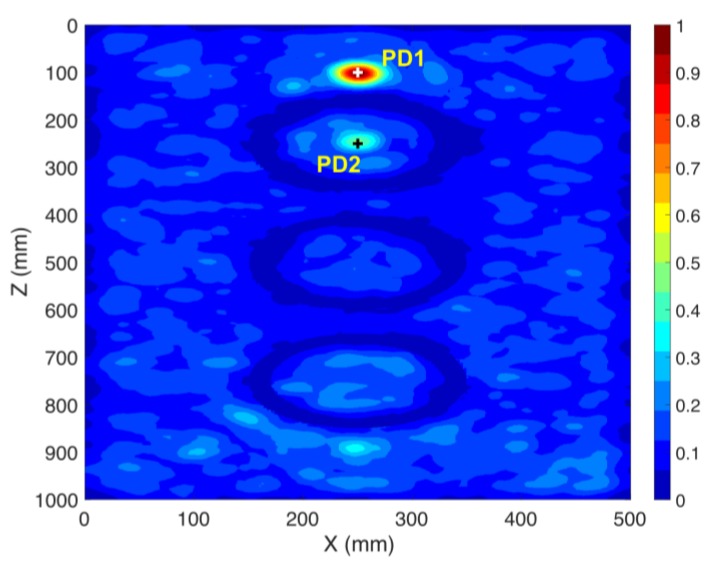
Normalized distribution of the maximum electric field power over the complete simulation time inside the transformer tank in the x-z cut plane (CS #5). The active parts of the transformer are included. The ground truth locations of the PD sources (PD1 and PD2) are shown by “+” markers. Only one sensor (S1) is used.

In practical cases, multiple sources might occur within the transformer tank. In this section, we show that the proposed method is able to locate multiple PD sources using only one sensor. To show this fact, we consider two case studies (CS #4 and CS #5). The location of the sensor (S1) is kept as that of cases CS #2 and CS #3. The locations of the PD sources are given in Table 1 and Table 2. In CS #4, PD1 and PD3 are considered, with PD1 located behind one of the active parts. In CS #5, we considered a more challenging scenario, in which PD1 and PD2 are considered, the latter being located in the middle of the active part (inside the cylindrical winding) of the transformer and the cylindrical winding acts as a barrier for the wave. TDoA approaches are not applicable to such a scenario, even with four sensors. Figure 6 and Figure 7 show the normalized distribution of the maximum electric field power over the whole simulation time inside the transformer tank in the x-z cut plane for CS #4 and CS #5, respectively. In both of the figures, the locations of the cylindrical windings can be clearly identified (corresponding to minimum field). The 3D location errors for CS #4 are estimated to be 5 and 6 mm for PD1 and PD3, respectively. The 3D location errors for CS #5 are estimated to be 4 and 3 mm for PD1 and PD2, respectively. It can be seen that the proposed method is capable of locating multiple simultaneous sources with high accuracy, considering the presence of the windings. It should also be noted that, in Figure 7, PD2 is located inside the winding and, as a result, its focal spot is less pronounced compared to PD1.

### 3.5. Further Discussion on the Performance of the Method 

**Figure 8:**
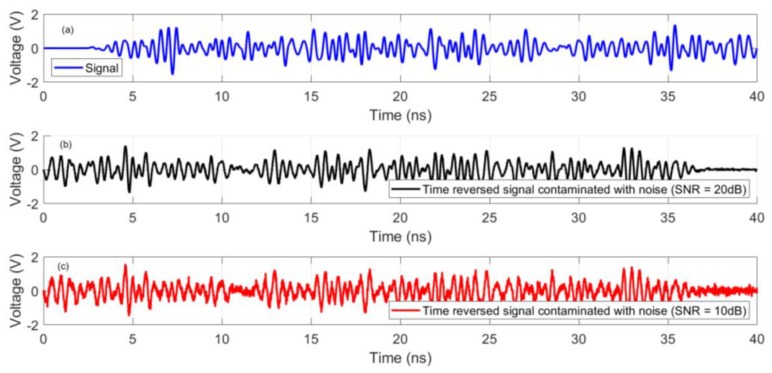
(**a**) Signal recorded by sensor S1. (**b**) The time reversed signal with added noise (SNR = 20 dB), and (**c**) the time reversed signal with added noise (SNR = 10 dB).

**Figure 9:**
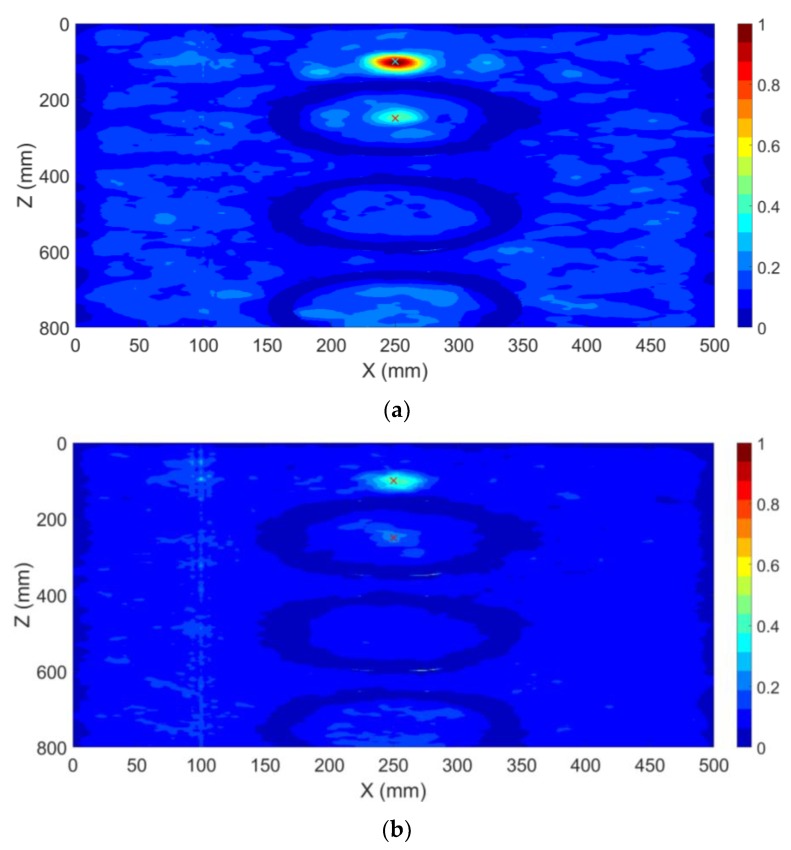
Normalized distribution of the maximum electric field power over the complete simulation time inside the transformer tank in the x-z cut plane (CS #5). (**a**) an SNR of 20 dB is considered and (**b**) an SNR of 10 dB is considered. The active parts of the transformer are included. The ground truth locations of the PD sources (PD1 and PD2) are shown by “+” markers. Only one sensor (S1) is used.

In order to further validate the applicability of the proposed method to localize PD sources, we investigated the effect of variations in length (5 mm, 10 mm, 20 mm, and 30 mm) and polarization ((0–90) degrees) of the PD source. No degradation in the accuracy of the proposed method was observed. Furthermore, to investigate the effect of the presence of environmental noise on the performance of the system, we added white Gaussian noise to the signal recorded by the sensor (S1) in CS #5. Two values were considered for the signal-to-noise ratio: SNR = 20 dB and SNR = 10 dB, where SNR is the Signal to Noise Ratio. Figure 8 shows the noiseless signal at the sensor along with the signal with added noise. Figure 9 shows the normalized distribution of the maximum electric field power over the complete simulation time inside the transformer tank in the x-z cut plane for CS #5 with added noise. Again, the cylindrical winding positions are clearly discernible. The 3-D location errors for CS #5 with added noise are estimated to be 4 and 3 mm for PD1 and PD2, respectively.

### 3.6. PD Localization in the Presence of the Transformer Core and Windings 

**Figure 10:**
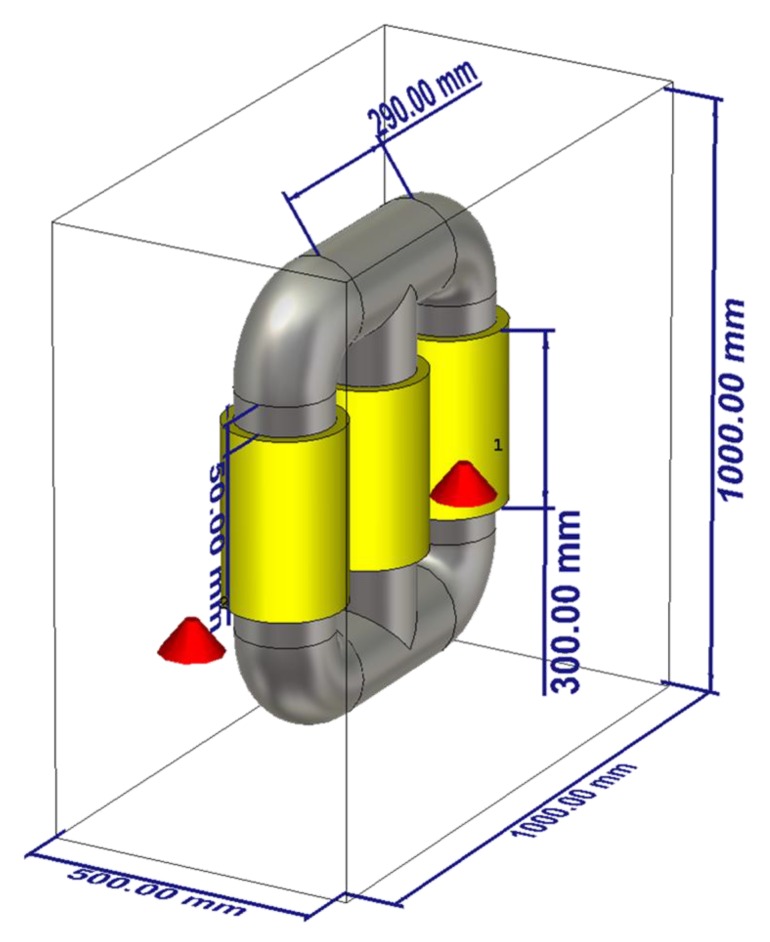
The geometry of the transformer tank including transformer windings and metallic core.

**Figure 11:**
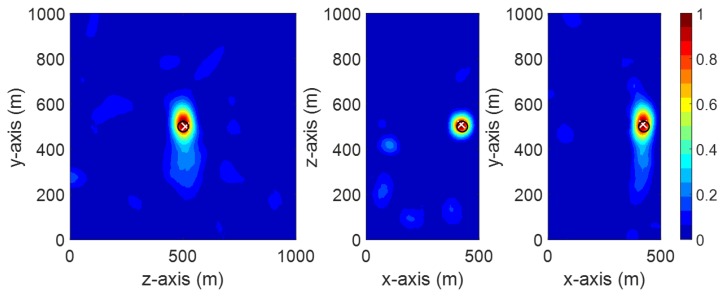
Normalized distribution of the electric field power at the optimal time slice provided by the entropy criteria.

Herein, to further evaluate the performance of the proposed method, we considered a more sophisticated model of the transformer including the windings and the core (see Figure 10). The transformer tank, the metallic core, and the transformer windings are assumed to be, respectively, steel (σ = 7.69e6 S/m), iron (σ = 1.04e7 S/m), and copper (σ = 5.8e7 S/m). The PD source was modeled as a dipole antenna excited with a Gaussian pulse of 0–2000 MHz bandwidth. The locations of the considered PD source (PD4) and the location of the sensor (S3) are given in Table 3. It should be noted that, in this section, use is made of the entropy criterion to obtain the optimal time slice of the time reversal process. Figure 11 shows the obtained electric field power distribution in the three cut planes at the optimal time slice. The localization error is about 14 mm.

### 3.7. Verification of the Simulation Scheme

**Figure 12:**
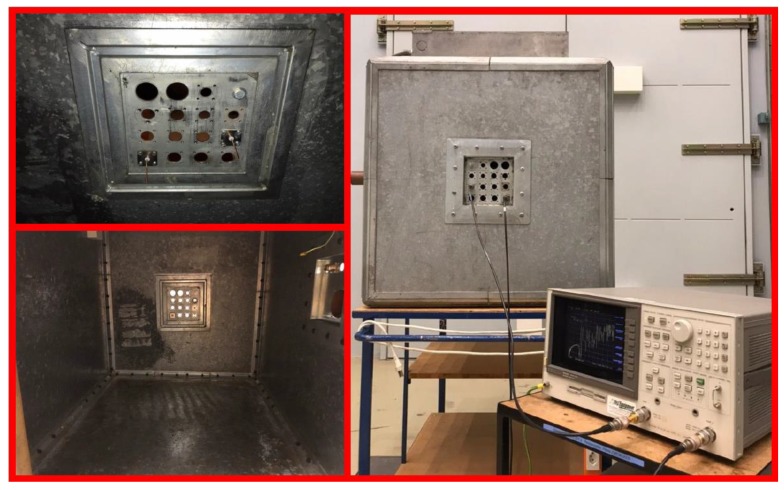
Experimental setup including the location of the antennas, the interior side of the cavity considered as the transformer tank, and the Vector Network Analyzer (VNA) that is used to measure the scattering parameters.

**Figure 13:**
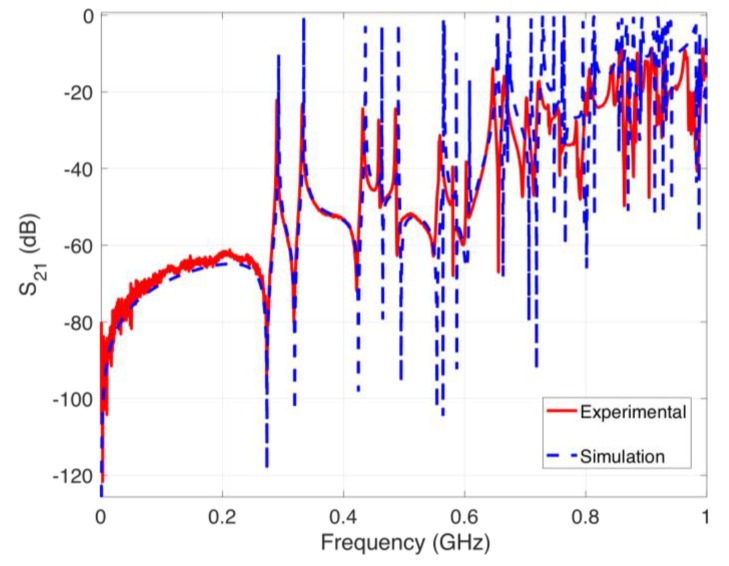
S21 parameter obtained using the experimental setup and the simulation scheme.

Here, we present a validation of our simulation scheme by comparing the simulated results with those obtained by measurement in the frequency domain. Our test setup (Figure 12) includes a rectangular tank with dimensions 970 × 730 × 730 mm, two monopole antennas with a length of 5.5 cm mounted on a wall of the tank, and a 9 kHz to 3 GHz Vector Network Analyzer (VNA). The first antenna is considered as a PD source and the second antenna is used as a sensor. Note that, although we perform our validation test in the frequency domain, the same results are expected in the time domain by applying an inverse Fourier transform. Figure 13 shows a comparison of the experimentally obtained S21 scattering parameter versus CST-MWS simulations. The observed results show good agreement between the CST-MWS and the VNA results for the transfer function (Fourier transform of the impulse response) from the source to the sensor in the linear, time-invariant medium. This lends support to the use of CST-MWS to simulate the transformer tank. We have not validated the TR location method experimentally at this time since the test requires equipment for the back-propagation phase that we do not have at our disposal at this time.

## 4. Conclusions 

In this work, we proposed a novel method to localize PD sources based on the concept of time reversal using both electromagnetic and acoustic waves. As a proof of concept, we presented here only UHF source localization results while the methodology is directly applicable to acoustic waves. To the best of our knowledge, this is the first time that the TR method is used to localize PD sources. Conventional methods such as TDoA require at least four sensors to locate PD sources unambiguously. The proposed method is able to reduce the number of required sensors for localization of PD sources to as few as a single sensor. In contrast with TDoA, the implementation of the proposed method with only one sensor does not require any time synchronization. The ability of the proposed method to locate multiple PD sources inside a transformer tank was demonstrated. Numerical simulations showed that multiple PD sources can be precisely localized with only one UHF probe. In the proposed method, the theoretical location accuracy is not affected by various factors such as the length and polarization of the PD sources, theoretically. The numerical simulations show that the maximum PD source localization error is less than 8 mm, which is lower than λ/10. The proposed method is suitable for factory acceptance tests (FAT) and site acceptance tests (SAT), as well as on-line diagnostic tools for power transformers, Gas Insulated Substations (GISs), and electric motors. 

This is the first time that electromagnetic TR is applied to locate PD sources in transformers. More in-depth theoretical and experimental investigations are needed to further assess the performance of the method on a real transformer and in the presence of noise.

## Figures and Tables

**Table 1:** List of the performed case studies and the resulting location error.

Case Studies	PD Source(s)	Sensors	Active Parts Incl. (Y/N)	Location Error (mm)
CS #1	PD1	S1, S2	N	6
CS #2	PD1	S1	N	5
CS #3	PD1	S1	Y	4
CS #4	PD1	S1	Y	6 (for PD1)
	PD3			5 (for PD3)
CS #5	PD1	S1	Y	3 (for PD1)
	PD2			4 (for PD2)

**Table 2:** Location of the considered UHF probes and PD sources inside the transformer tank.

Port	Location [x, y, z]
PD1	[250, 250, 100]
PD2	[250, 250, 250]
PD3	[150, 250, 100]
S1	[100, 50, 900]
S2	[350, 0, 900]

**Table 3:** Location of the considered sensor and PD source inside the transformer tank, including the transformer winding and metallic core.

Port	Location [x, y, z]
PD4	[425, 500, 500]
S3	[200,100, 950]
